# Plexiform Ameloblastoma

**DOI:** 10.5005/jp-journals-10005-1140

**Published:** 2012-02-24

**Authors:** Sreelalita Celur, K Sunil Babu

**Affiliations:** Reader, Department of Oral and Maxillofacial Surgery, MNR Dental College, Sangareddy, Andhra Pradesh, India; Reader, Department of Pedodontics and Preventive Dentistry, Mamata Dental College, Khammam, Andhra Pradesh, India, e-mail: drsunil16@yahoom.com

**Keywords:** Ameloblastoma, Plexiform, Costochondral graft, Mandible rehabilitation

## Abstract

The ameloblastoma is a benign but aggressive neoplasm of odontogenic origin. However, no enamel or hard tissue is formed by the tumor cells. Ameloblastomas are infamous for their invasive growth and their tendency to recur. Robinson (1937) as a benign tumor that is ‘usually unicentric, nonfunctional, intermittent in growth, anatomically benign and clinically persistent.’ They may occur at any age, even though nearly half of the tumors do occur between the ages of 20 and 40 years. This is the most common neoplasm affecting the jaws, yet only accounts for 1% of all tumors of the maxilla and mandible and 11% of all odontogenic tumors.

This report presents a case of ameloblastoma involving entire ramus and part of body of mandible with resorption of the mesial and distal root apices of second molar and distal root of mandibular first molar. The lesion extending till the base of mandible surrounding the crown of the unerupted third molar resembling the dentigerous cyst. This was surgically resected followed by harvesting the contralateral sixth costochondral rib graft.

**How to cite this article:** Celur S, Babu KS. Plexiform Ameloblastoma. Int J Clin Pediatr Dent 2012;5(1):78-83.

## INTRODUCTION

The ameloblastoma is a true neoplasm of enamel organ- type tissue which does not undergo differentiation to the point of enamel formation. It is described by Robinson (1937)^1^ as a benign tumor that is ‘usually unicentric, nonfunctional, intermittent in growth, anatomically benign and clinically persistent.’ They may occur at any age, even though nearly half of the tumors do occur between the ages of 20 and 40 years. This is the most common neoplasm affecting the jaws, yet only accounts for 1% of all tumors of the maxilla and mandible and 11% of all odontogenic tumors.^2^ It is an aggressive benign tumor of epithelial origin which may arise from the enamel organ,^3^ follicle, periodontal ligament,^4^ and lining of an odontogenic (dentigerous) cyst^3,4^ or the marrow of the jaws. Ameloblastomas are infamous for their invasive growth and their tendency to recur. Small incipient lesions may be mistaken for a common periapical granuloma or cyst and the tooth may be treated endodontically or extracted with the lesion going undiagnosed or inadequately treated. Differential diagnosis prior to definitive treatment is mandatory and necessitates a tissue biopsy as this lesion requires to be treated more aggressively than other benign periapical lesions. This case report presents a study of a plexiform ameloblastoma with surgical management.

## CASE REPORT

**Fig. 1:**
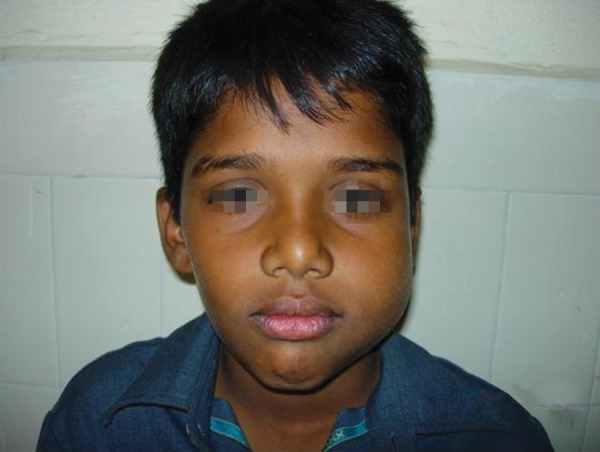
Facial asymmetry and a firm swelling in the area of the left mandibular ramus region extending to the base of the mandible

**Fig. 2:**
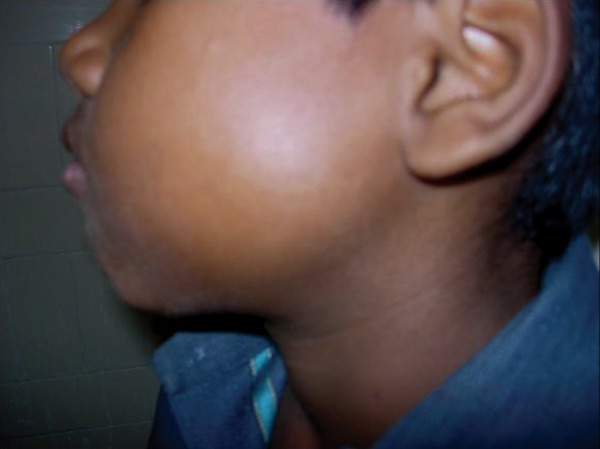
A firm swelling in the area of the left mandibular ramus region extending to the base of the mandible

A 13-year-old male presented with a small swelling in the vestibular area of the left second mandibular molar region. Extraoral clinical examination of the patient was notable for facial asymmetry and a firm swelling in the area of the left mandibular ramus region extending to the base of the mandible (Figs 1 and 2). Intraorally, the area was slightly tender and the tooth had grade-1 mobility. There was no nerve deficit or adenopathy in the head and neck. Vitality testing of the tooth revealed the tooth to be vital.

**Fig. 3:**
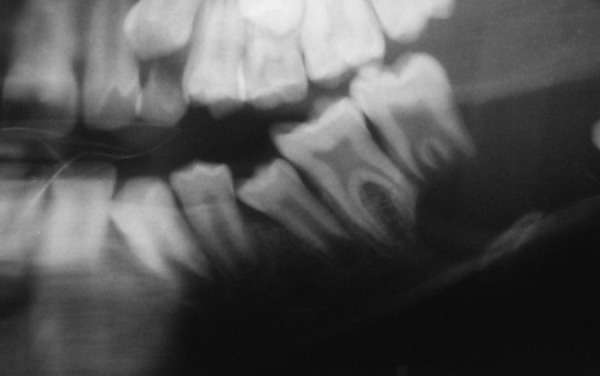
Radiograph showing radiolucency extending till the base of mandible surrounding the crown of the unerupted third molar resembling the dentigerous cyst

Radiographically, solitary radiolucent lesion involving entire ramus and part of body of mandible with resorption of the mesial and distal root apices of second molar and distal root of mandibular first molar. The lesion extending till the base of mandible surrounding the crown of the unerupted third molar resembling the dentigerous cyst (Fig. 3). The medical history was not significant and the patient was in good general health.

**Fig. 4:**
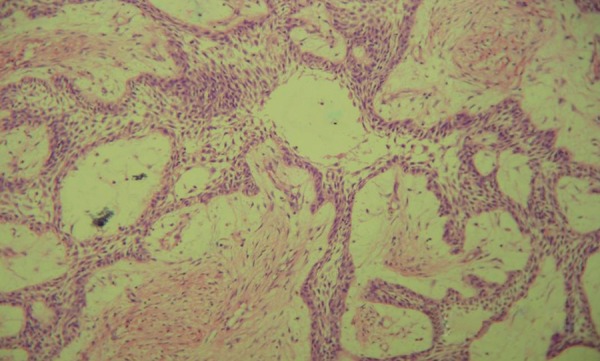
Histological slide confirmed the diagnosis of a plexiform ameloblastoma

Incisional biopsy was done under local anesthesia and sent for histopathological examination. The pathology report confirmed the diagnosis of a plexiform ameloblastoma (Fig. 4) and the patient was scheduled for surgical resection of the involved mandible followed by placement of the costochondral graft.

**Fig. 5:**
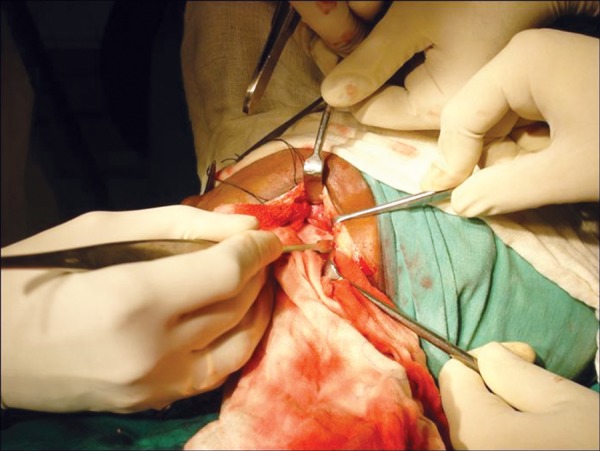
Extraoral incision and retraction of the left mandibular ramus region

**Fig. 6:**
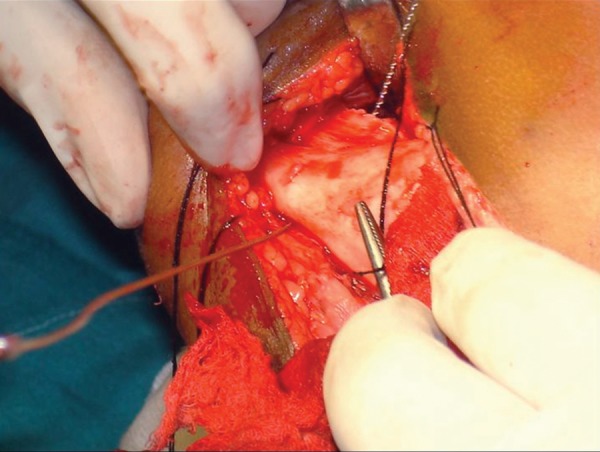
Sectioning of the mandible

**Fig. 7:**
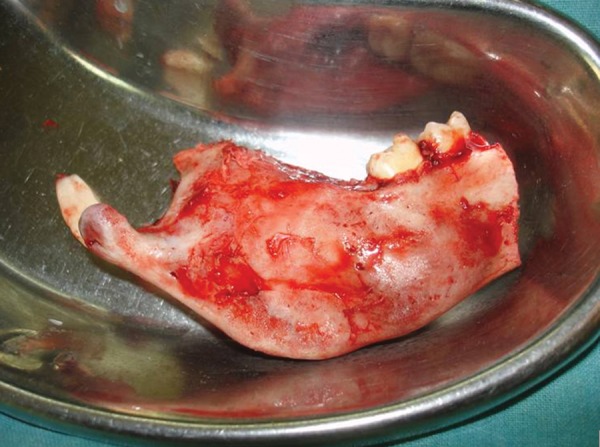
Sectioned mandible maintaining a safe margin of 1.5 mm of uninvolved bone

**Fig. 8:**
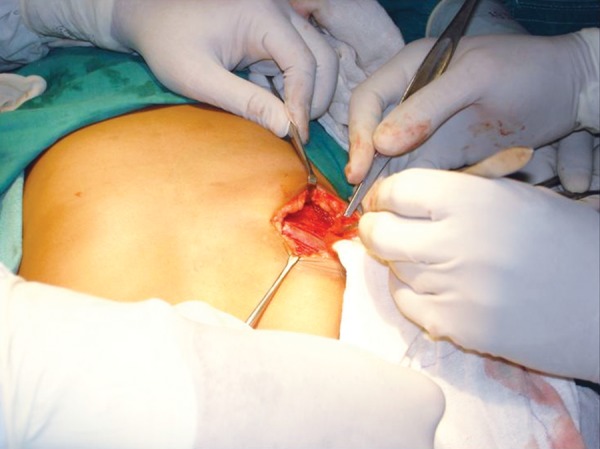
Contralateral sixth rib was harvested as a costochondral graft through inframammary incision

**Fig. 9:**
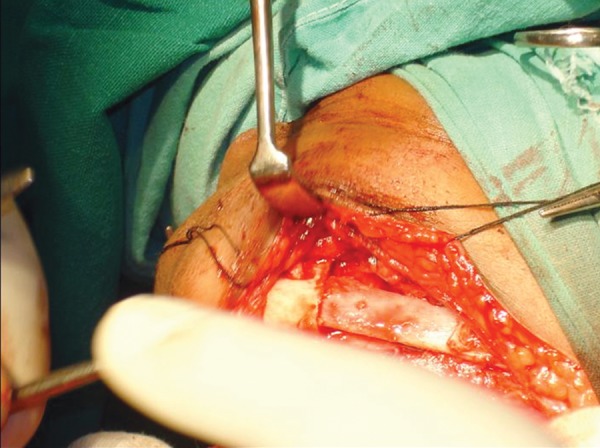
Costochondral graft secured into place in continuation with the distal end of resected mandible

**Fig. 10:**
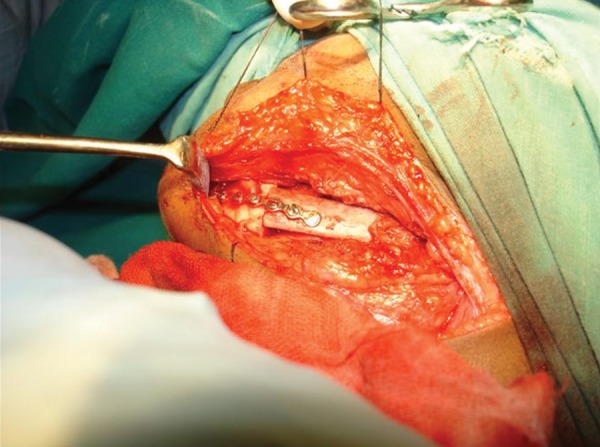
Costochondral graft secured into place by means of reconstruction plate

Surgical resection of the tumor was carried out through an extraoral submandibular approach. Premolars were extracted and mandible was sectioned maintaining a safe margin of 1.5 mm of uninvolved bone (Figs 5 to 7). Temporary maxillomandibular fixation was done. Contralateral sixth rib was harvested as a costochondral graft through inframammary incision and secured into place by means of reconstruction plate (Figs 8 to 10).

**Fig. 11:**
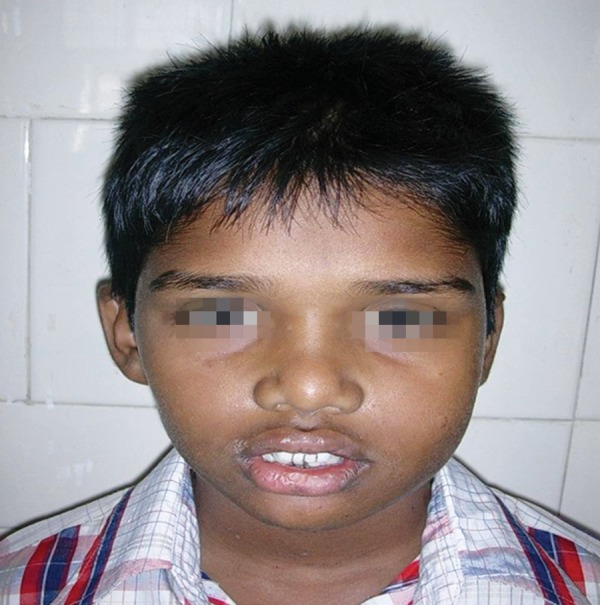
After a week, patient was recalled for the check-up

**Fig. 12:**
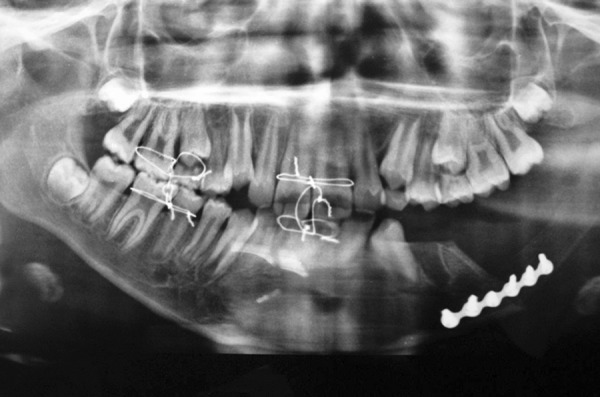
Radiograph to ensure the stability of the graft

After a week-patient was recalled for the check-up, a radiograph was taken to ensure the stability of the graft (Figs 11 and 12).

## DISCUSSION

The ameloblastoma is a benign but aggressive neoplasm of odontogenic origin. However, no enamel or hard tissue is formed by the tumor cells.^5^ It comprises 1% of all radiolucent jaw lesions.^6^ Ameloblastomas arise from either neoplastic transformation of odontogenic cyst epithelium or from residual epithelial rests left over from the formation of teeth, such as remnants of the enamel organ (reduced enamel epithelium) found over the crown of an unerupted tooth, remnants of Hertwig’s epithelial root sheath (rests of Malassez) in the periodontal ligament or remnants of the dental lamina (rests of Serres). Ameloblastomas may be confused clinically with other jaw lesions, and occasionally with infiltrating neoplasms of the maxillary sinus, particularly those of salivary gland origin.^5^

## CLINICAL PRESENTATION

The age range for ameloblastomas is from 20 to 50 years^7-9^with the average age being 27.18. There is no sex or racial predilection. Eighty percent of ameloblastomas occur in the mandible—almost exclusively in the molar ramus region of the mandible^10-12^ and are often associated with an unerupted teeth.^13^ The remaining 20% occur in the maxilla with the maxillary tuberosity being the most common site.^3^Ameloblastomas are nonencapsulated tumors.

Histologically, there are two basic patterns, follicular and plexiform with the former being more common. In both patterns the stroma is composed of mature fibrous connective tissue; however, the follicular form contains islands of epithelial elements within the stroma and the plexiform contains cords of epithelial elements within the stroma.^3^ The common denominator to all ameloblastomas is well-differentiated palisaded cells found around the periphery of nests, strands and networks of epithelium. Nuclei of the palisaded cells are typically polarized away from the basement membrane. Budding of epithelium from these proliferative nests and strands is also characteristic of this lesion. Palisading cells and budding epithelium are found in all microscopic subtypes (follicular, cystic, plexiform, desmoplastic, acanthomatous, granular cell).^5^

Ameloblastomas are classified into four groups; unicystic, solid or multicystic, peripheral and malignant. The unicystic ameloblastoma is essentially a ‘cystic’ lesion with either an intraluminal or an intramural proliferation of the cystic lining.^14^ Radiographically, it is a well- circumscribed slow-growing radiolucency. Microscopically, it is a ‘cystic’ lesion with three significant features as described by Leider et al:

1. Columnar cells resembling ameloblasts occupy the basal cell layer
2. Hyperchromatic nuclei having a vacuolated atypical cytoplasm are polarized from the basal lamina and
3. A loose stellate reticulum-like epithelium lining the basilar ameloblasts.^15^ Unicystic ameloblastomas are less aggressive than solid ameloblastomas.^14^

The multicystic or solid ameloblastoma can grow to an enormous size. It can infiltrate into adjacent structures including intracranial sites, and has the ability to recur and even metastasize. It occurs in a slightly older age group than its unicystic counterpart. The majority of the cases involve the mandible, but the maxilla can also be involved. Radiographically, the appearance is variable with the exception of the desmoplastic variant, but it is generally unilocular or multilocular.^16^ Microscopically, this lesion can exhibit a variety of patterns. Multicystic ameloblastoma has a poorer prognosis than the cystic lesion.^16^

The peripheral ameloblastoma is the soft tissue version of the central intraosseous ameloblastoma. It occurs in the alveolar mucosa; however, the underlying bone can be involved due to secondary erosion.^17^ This type of tumor is rare.

The malignant ameloblastoma is also a rare entity. Elzay and Corio et al defined this lesion as an ameloblastoma that has metastasized but still maintains its classical microscopic features.^18,19^ Clinically, the majority of patients (75%) present with a chief complaint of a slow growing painless swelling. Signs and symptoms may include; facial deformity, swelling (75%), pain (33%), malocclusion, loosening of teeth, ill-fitting dentures and bridges, ulcerations and periodontal disease.^20^

Radiology and location of ameloblastomas are key factors to a correct diagnosis. Plain radiography, panoramic radiographs, conventional tomography (CTs), and magnetic resonance imaging (MRIs) are all used as diagnostic aids. Findings may include expansion of cortical plate with scalloped margins, multiloculations or ‘soap bubble’ appearance and/or root resorption. CTs are used to delineate soft tissue masses, destruction of cortical bone and extension of the tumor into adjacent structures. MRIs, even though not useful for hard tissue examination, are used to provide information regarding edge definition and tumor consistency.^21^

Ameloblastoma is a locally benign invasive tumor that has a high tendency to recur, metastasize and even undergo malignant transformation. It has a high recurrence rate if not adequately removed^22^ but local recurrence may occur even in patients who have undergone satisfactory primary surgical treatment.^23^ As these tumors recur they become more aggressive and can develop into a lesion that is more aggressive than a sarcoma.^20^ Recurrence seems to depend on several factors, such as (1) method of treatment of the primary lesion, (2) the extent of the lesion and (3) the site of origin.^24^

Among the various types of ameloblastomas there are differences in recurrent patterns. Multicystic ameloblastoma has a much higher rate of recurrence than unicystic ameloblastoma. The reason for this higher rate is believed to be because of the numerous microextensions the tumor has projecting into the bone.^25,26^ Gardner and Pecak stress that the type of treatment required is highly dependent on the type of ameloblastoma present.^22^

## MANAGEMENT

Proper diagnosis and management of ameloblastomas cannot be overemphasized because of the high recurrence rate demonstrated by this lesion. Ominous signs, such as root resorption and tooth mobility should not go unnoticed. Paresthesia which may be prevalent in larger lesion should also raise suspicion as to an aggressive or malignant lesion. It is imperative therefore, that, after clinical and radiographic evaluation of a patient with a periapical lesion, diagnosis be confirmed by obtaining a specimen and sending it for microscopic examination (even if the lesion is small and or associated with a pulpal-involved tooth) so that prompt appropriate treatment may be rendered.

An incisional or excisional biopsy may be done depending on the size of the lesion and its clinical features.^16^An incisional biopsy is advantageous if a representative specimen can be obtained. This will provide the clinician with a definitive diagnosis and allow for an appropriate workup before developing a therapeutic protocol.

An exception is a patient with a small, unilocular lesion in which the clinical impression is an odontogenic cyst or fibro-osseous lesion where an excisional biopsy is usually performed.^16^

If subsequent microscopic examination confirms an ameloblastoma, the clinician must then decide on additional surgery and take the necessary measures.

Surgery is the mainstay of therapy for ameloblastomas today. Treatment ranges from conservative surgery to more radical procedures. Conservative therapy includes radiotherapy, curettage and enucleation.

Recurrence rates also vary for the different procedures used to treat the primary lesion. Several authors have found a recurrence rate of 55 to 90% for all ameloblastomas treated conservatively (enucleation and curettage).^11^ However, the incidence of recurrence following radical resection is 5 to 15%.24,25

Radical surgery, as defined by Muller and Slootweg, is a procedure in which the ameloblastoma is removed with a margin of ‘normal bone’.^10^ Most investigators believes in resecting at least 1 cm of normal bone beyond the tumor margin.^22,27^ When the tumor has perforated bone, removal of adjacent soft tissue extending to the next adjacent anatomic boundary must be performed to ensure complete tumor-free soft tissue margins.^16^

Preservation of the inferior alveolar nerve is not prudent when it is involved by the tumor, and it should be resected en bloc with the specimen in such cases. This may have been the cause of the recurrence in our case. Immediate nerve reconstruction after extirpation of the tumor restores lower lip sensation for the patient. It is most important to emphasize, to both the clinician and the patient, the need for a definitive treatment protocol and lifetime periodic follow-up for detection of recurrence as even a 5 year tumor- free period does not necessarily mean a cure.^5^

An ameloblastoma is an epithelial tumor similar to a basal cell carcinoma histologically. Therefore, some investigators contend that their radiosensitivities must also be similar.^28^ However, radiation therapy is rarely used as a primary treatment. Gardner believes that radiotherapy should only be used for inoperable cases.^20^ Other investigators advocate that radiotherapy in conjunction with surgery may have a place in the management of selected patients with recurrence. Pinsolle et al believe that surgery and radiotherapy (50 Gy postoperatively) should be used for (1) mandibular recurrences when the first surgical treatment was adequate, (2) for all recurrences and (3) when soft tissue involvement or positive surgical margins are present after a wide resection.^28^

## CONCLUSION

This case report illustrates the importance of adequate radical resection to avoid recurrence. All efforts must be made to excise the recurrent lesion because death can occur as a result of damage to vital structures by massive local disease and metastasis. Recurrences often present after 15 years or more.^22^ Therefore, it is important to emphasize the need for long-term periodic follow-ups.
